# The Relationship Between Anthropometric Z-Score Measurements and Ocular Structures in Turkish Children

**DOI:** 10.22336/rjo.2023.60

**Published:** 2023

**Authors:** Erkan Bulut, Ahmet Kürşad Sakallioğlu, Özlem Dayi, Goksu Alacamli

**Affiliations:** * Department of Opticianry, Gelişim University, Vocational School of Health Services, Istanbul, Turkey; ** Department of Ophthalmology, Trakya University, School of Medicine, Edirne, Turkey; *** Department of Ophthalmology, Beylikdüzü Public Hospital, Istanbul, Turkey

**Keywords:** anthropometric Z-score values, ocular parameters in children, Z-score, body mass index, spherical equivalent refraction

## Abstract

**Objective (Aim):** This study explores the contact between anthropometric Z-score values and ocular parameters in children. Recent studies investigated the relationship, and contact between anthropometric measurements and ocular parameters in children, and height, weight, body mass index, and percentile curves are mostly used as anthropometric data. However, today, different scoring systems such as “Z-score” classify anthropometric values.

**Methods:** Height and body mass index Z-scores were calculated for 725 children. Biometric and refractive measurements of all children were noted. For different reference values, those with a Z-score below the negative value of the reference were defined as a low Z-score, those between the negative and positive value of the reference were defined as a normal Z-score, those with a Z-score greater than the positive value of the reference were defined as a high Z-score. The mean ocular measurement results in the low, normal, and high Z-score groups were compared, and they were pointed to reach the reference value in both negative and positive sides which created the foremost critical contrast between the groups.

**Results:** For a value of “-1” and “+1.5” in the height Z-score, from low to normal and from there to high Z-score group, axial length, and average corneal radius increased, and average corneal power decreased significantly. Anterior chamber depth increased from normal to high Z-score group, but no critical distinction was made between low and normal Z-score groups. Moreover, no critical distinction was observed in spherical equivalent refraction, central corneal thickness for height, and all values of body mass index.

**Conclusion:** Considering a Z-score value of “-1” and “+1.5” as a reference value in children and anticipating the changes that may happen in the ocular structures of children at both ends of the Z-score, it may be useful to understand the effect of body development on ocular development more.

**Abbreviations:** AL = Axial Length, ACD = Anterior Chamber Depth, BMI = Body Mass Index, NCHS = The United States National Center for Health Statistics, WHO = World Health Organization, NFHS 2 = National Family Health Survey 2, SER = Spherical Equivalent Refraction, CR = Average Corneal Radius, CP = Average Corneal Power, CCT = Central Corneal Thickness, HFAsZ = Height for Age Z-Score, BMIsZ = BMI for Age Z-Score, L = Low Z-score, N = Normal Z-score, H = High Z-score

## Introduction

Critical anthropometric changes in tallness (height) and weight estimations were detected in newborn children, children, and teenagers (adolescents) matured amid the final four decades. In all the age bunches, both boys and young ladies were taller and heavier than their peers from the 1960s [**1**]. Also, numerous studies have previously mentioned the contact between anthropometric measurements and the dimensions of the ocular globe. The effect of height on the axial length (AL) of the eye was examined in these studies, and it was observed that taller individuals had a greater likelihood of having a longer globe AL, higher anterior chamber depth (ACD), thinner lenses, and flatter corneas [**2**]. 

The effects of weight on ocular structures have also been discussed in recent studies. Particularly, an affiliation of obesity with a few ocular maladies including glaucoma, diabetic retinopathy, cataract, and age-related macular degeneration has been specified [**3**,**4**]. Obesity has also been shown to affect anterior chamber parameters. ACD and anterior chamber angle decrease in individuals with increased body mass index (BMI) [**5**].

The studies investigating the relationship between anthropometric measurements and ocular parameters in children mostly used height, weight, body mass index, and percentile curves as anthropometric data [**6**-**9**]. However, today, there are also different scoring systems such as the “Z-score” that classify anthropometric values.

In 1975, the United States National Center for Health Statistics (NCHS) recommended expressing deviations from the reference median’s anthropometric measurements in terms of standard deviation or Z-scores [**10**]. The NCHS/World Health Organization (WHO) growth reference curves were converted to Z-score format and used to estimate the prevalence of malnutrition in preschool-aged children. The Z-score indicates how widely spread the data in a specific population is from the reference median [**11**]. Positive scores indicate values above the population, and negative values indicate values below the population. The amount of deviation (in the positive or negative direction) of the Z-score from 0 is specifically relative to the amount of deviation from the mean [**12**]. A value of 2 standard deviations (the 97.7th percentile) above the mean corresponds to a Z-score of +2.0, while a value of 2 standard deviations below the mean corresponds to a Z-score of -2.0. The mean of 0 and the normal ranges of -2.0 to +2.0 values make the clinical interpretation simple [**13**]. The Z-score with percentile equivalence and the WHO classification of nutrition status in children and adolescents based on anthropometry are summarized in **Table 1** and **Table 2** respectively [**14**].

**Table 1:** Z-score with percentile equivalence

Z-score value	Percentile equivalence
-3	0.1
-2	2.3
-1	15.9
+1	84.1
+2	97.7
+3	99.9

**Table 2:** The WHO (World Health Organization) classification of nutrition status in children and adolescents based on anthropometry

Classification	Condition	Age: 0-60 months indicator and cut-off	Age: 61 months - 19 years indicator and cut-off
Based on body mass index	Possible risk of overweight	BMI-for-age (or weight-for-height) >1SD	
	Overweight	BMI-for-age (or weight-for-height) >2SD	BMI-for-age >1SD (equivalent to BMI 25 kg/m2 at 19 y)
	Obese	BMI-for-age (or weight-for-height) >3SD	BMI-for-age >2SD (equivalent to BMI 30 kg/m2 at 19 y)
	Thin		BMI-for-age < -2 to -3 SD
	Severely thin		BMI-for-age <-3 SD
Based on weight and height	Stunted	Height-for-age <-2SD to -3SD	Height-for-age <-2SD to -3SD
	Severely stunted	Height-for-age <–3SD	Height-for-age <–3SD
	Underweight	Weight-for-age <-2SD to -3SD	Weight-for-age (up to 10y) <-2SD to -3SD
	Severely underweight	Weight-for-age <–3SD	Weight-for-age (up to 10y) <–3SD
	Wasted	Weight-for-height <-2SD to -3SD	
	Severely wasted	Weight-for-height <–3SD	
BMI = Body mass index; SD = standard deviation; kg = kilogram; y = years			

National Family Health Survey 2 (NFHS 2) uses Z-scores to grade undernutrition and the use of the Z-score is widely recommended [**15**]. Once the new recommended Z-score system is used to evaluate the link between anthropometric and ocular measurement data, the new results may show a modern point of view on the ocular measurement of the patients. In this way, the main goal of this present study is to detect the association or the contact, relationship between anthropometric values Z-score and ocular parameters in children, as well as the impact of anthropometric values Z-score on visual parameters. 

## Materials and methods

*Data collection and exclusion criteria*

This prospective observational case series was conducted on 725 eyes of 725 healthy children who were admitted to the Ophthalmology Department for routine ophthalmological examination between November 2019 and March 2023. Patients older than 15 years of age or with any systemic disease affecting growth and body development, patients under any regular medication, patients who had any ocular disease other than refractive disorders, patients whose optimum ophthalmologic examination and measurements could not be performed due to patient incompatibility were not included in the study.

The sex of the subjects (patients), age at the time of examination (months), height (centimeters), and weight (kilograms) were noted. Refractive errors in both eyes of the patients were measured using the auto-refractometer device (Topcon KR-800, Topcon Medical Systems Inc., Fukuoka, Japan). Spherical equivalent refraction (SER) values were computed according to the formula: spherical diopters + (cylinder diopters/2) and noted. A biometry device (Nidek Axial Length-Scan, Nidek CO., Gamagori, Japan) was used for biometric measurements of both eyes of the patients such as AL, ACD, average corneal radius (CR), average corneal power (CP), and central corneal thickness (CCT). CR and CP data (values) were computed according to the arithmetic mean of the values in flat and steep corneal keratometric quadrants. 

During biometric measurements, a concern that accommodation in different strengths may adversely affect the measurement results arose. Hughes et al. [**16**] recently reported in their study that reliable biometry measurements and active accommodation were observed for 76 of 87 participants who were included in their analysis. LT and anterior segment length increased, and ACD and vitreous chamber depth decreased significantly with increasing accommodation. Also, Read et al. [**17**] reported that the eye experiences considerable axial elongation during a short time of accommodation, and the amount of this change in eye length increases with increased accommodation demands. Owing to these outcomes, to prevent errors in the biometric measurement results due to the accommodation that may occur at different times and different strengths, all measurements were performed and noted under cyclopentolate hydrochloride drop (Sikloplejin 1%, Abdi Ibrahim Pharmaceuticals, Turkey) administration at a concentration of 1% into both eyes.

Height and weight measurements of patients were taken using a digital scale and a wall-mounted Harpenden stadiometer. The BMI was measured using the weight formula in kilograms divided by the square of the height in meters (kg/m²). For Z-score calculations, the formula of dividing the outcome by the standard deviation by subtracting the mean predicted measurement according to age and sex from the patient’s measurement value was used. The gender, age, height, and weight information of children younger than 60 months were entered into the WHO Anthro software (version 3.2.2) [**18**]. The same information for children older than 60 months was entered into the WHO AnthroPlus software (version 1.0.4) which was created for patients older than 60 months [**19**]. The results of height for age Z-score (HFAsZ) and BMI for age Z-score (BMIsZ) were obtained from the software.

To determine the differences in ocular parameters of the patient groups with different Z-score values, the patients were divided into 3 groups in terms of HFAsZ and BMIsZ. To observe which Z-score value would make a difference in terms of ocular biometric data, negative/positive 1, 1.5, and 2 Z-score values for these 3 groups were taken as reference for both HFAsZ and BMIsZ. For example, for a value of “1”, the group of patients with a low Z-score (L) consisted of patients with a Z-score less than -1; the group of patients with a relatively normal Z-score (N) consisted of patients with a Z-score between -1 and +1, including -1 and +1; the group of patients with a high Z-score (H) consisted of patients with a Z-score greater than +1. Similarly, for a value of 1.5, the L consisted of patients with a Z-score of less than -1.5; N consisted of patients with a Z-score between -1.5 and +1.5 inclusive; H consisted of patients with a Z-score greater than +1.5. For a value of 2, the L consisted of patients with a Z-score of less than -2; N consisted of patients with a Z-score between -2 and +2 inclusive; H consisted of patients with a Z-score greater than +2.

After examining the comparison of ocular biometric measurements between groups for Z-score values of 1, 1.5, and 2, a new group was created for both HFAsZ and BMIsZ based on the results obtained. In this group, patients with a Z-score below -1 were arranged as L, N consisting of patients with a Z-score between -1 and +1.5 inclusive, and those above +1.5 as H.

*Ethical approval*

The research complied with all relevant national regulations and institutional policies and followed the tenets of the Helsinki Declaration. Informed consent was obtained from the parents and legal guardians of the patients after a clarification of the nature and conceivable results of the study. The study was approved by the Ethics Committee of Beylikdüzü Public Hospital, Istanbul, Turkey (2057/22.11.2019).

*Statistical Analysis*

Statistical analysis was performed using IBM SPSS Statistics (SPSS Inc., Chicago, IL) Version 20.0 for Windows. Numerical descriptive statistics were presented as means and standard deviations for normally distributed variables. Descriptive statistics of categorical variables were presented as a percentage. Kolmogorov Smirnov test was performed to assess the normality of data. An independent sample t-test was performed to compare independent groups. A One-way ANOVA test was used to compare numerous groups with each other. The relationship between the numerical variables was surveyed with the Pearson correlation test. The correlation coefficients were interpreted as follows: less than 0.4, weak; 0.4-0.6, moderate; 0.6-0.8, strong and 0.81-1.00 high strong relationship [**20**]. The significance level was p<0.05.

## Results

This prospective observational case series was conducted on 392 (54.1%) females and 333 (45.9%) males of a total of 725 patients. The mean ages of all, female, and male patients were 101.6±30.8 (minimum 36, maximum 178), 103.1±29.4 (minimum 40, maximum 156), and 99.8±32.3 (minimum 36, maximum 178) months respectively. Also, mean HFAsZ for all, female, and male patients were 0.1±1.9 (minimum -4.1, maximum 3.1), 0.4±1.0 (minimum -2.0, maximum 3.8), 0.2±1.2 (minimum -4.1, maximum 3.13); and mean BMIsZ for all, female, and male patients were 0.7±1.3 (minimum -6.0, maximum 4.7), 0.6±1.1 (minimum -2.5, maximum 3.1), 0.7±1.4 (minimum -6.0, maximum 4.7) respectively. No statistically significant differences between male and female patients in terms of age, HFAsZ, and BMIsZ (respectively; p=0.151, p=0.054, p=0.090) were observed.

Statistical differences were insignificant between both eyes of patients in terms of SER, AL, ACD, CP, CCT, and CR values (respectively, p=0.583, p=0.921, p=0.567, p=0.721, p=0.641, p=0.966). Due to the lack of significant difference in biometric measurements between the two eyes of the patients, to avoid bias, the right eyes of all patients were used as a reference for the statistical comparisons.

Comparing the ocular measures of male and female subjects (patients), the AL, ACD, CR, and CCT measurement values were higher in male patients, whereas the CP measurement values were considerably higher in female subjects (patients). Male and female patients did not shift statistically significantly in terms of SER and CCT. The mean values of the SER, AL, ACD, CR, CP, and CCT are presented in **Table 3**.

**Table 3:** Comparison of the ocular measurements between female and male patients

	Male			Female			
Meas.	Mean±SD	Max	Condition	Mean±SD	Min	Max	P value
SER	0.25±2.50	-6.63	7.13	0.58±2.56	-6.13	7.38	0.081
AL	23.32±1.13	19.91	26.18	22.76±1.29	20.09	30.92	*<0.001
ACD	3.83±0.27	3.08	4.55	3.72±0.22	2.93	4.29	*<0.001
CR	7.82±0.23	7.25	8.53	7.71±0.26	6.85	9.61	*<0.001
CP	43.19±1.29	39.57	46.57	43.86±1.41	40.32	49.31	*<0.001
CCT	571.23±35.6	465	643	565.00±35.2	479	691	*0.039
Meas = measurement, SD = standard deviation, Min = minimum, Max = maximum, SER = spherical equivalent refraction, AL = axial length, ACD = anterior chamber depth, CR = average corneal radius, CP = average corneal power, CCT = central corneal thickness. *Statistically significant value							

The Pearson correlation test showed a positively strong correlation between the patient’s height and weight (r=0.856, p<0.001). 

In groups created according to the value of “1” on the positive and negative sides for HFAsZ; L, N, and H included 104, 482, and 129 patients, and for BMIsZ, L, N, and H included 50, 396, and 279 patients respectively. Likewise, for the value of “1.5” for HFAsZ; L, N, and H included 54, 604, and 67 patients, and for BMIsZ, L, N, and H included 20, 526, and 179 patients respectively. For the value of “2” for HFAsZ; L, N, and H included 14, 686, and 25 patients, and for BMIsZ, L, N, and H included 18, 590, and 117 patients respectively. In the final value of “-1 to +1.5”; L, N, and H included 104, 554, and 67 patients for HFAsZ, and L, N, and H included 50, 496, and 179 patients for BMIsZ respectively.

**Table 4** and **Table 5** respectively show the measurement data for both HFAsZ and BMIsZ in each group for the values of “1”, “1.5”, and “2” and the final group for the value of “-1 to +1.5”. The statistical comparison of these measurement results between the groups is shown in **Table 6**. 

**Table 4:** The measurement values of L, N, and H for HFAsZ for the values of “1”, “1.5” and “2” and the final group “-1 to +1.5”

		HFAsZ values for ±1			HFAsZ values for ±1.5			HFAsZ values for ±2			HFAsZ values for -1 to +1.5		
Meas.	Gr.	Mean±SD	Min	Max	Mean±SD	Min	Max	Mean±SD	Min	Max	Mean±SD	Min	Max
SER	L	0.75±2.72	-4.75	7.37	0.44±3.04	-4.75	7.37	0.75±3.14	-3.25	7.12	0.75±2.72	-4.75	7.37
	N	0.56±2.44	-6.25	7.25	0.48±2.45	-6.25	7.25	0.42±2.50	-6.50	7.37	0.42±2.46	-6.25	7.25
	H	-0.26±2.59	-6.62	6.62	-0.01±2.82	-6.62	5.75	0.44±3.14	-6.62	5.75	-0.01±2.82	-6.62	5.75
AL	L	22.69±1.265	19.91	24.74	22.84±1.43	19.91	25.74	22.65±1.48	19.91	24.55	22.69±1.26	19.91	25.74
	N	22.98±1.21	20.32	30.92	22.98±1.21	20.32	30.92	23.01±1.24	20.09	30.92	23.01±1.23	20.32	30.92
	H	23.42±1.29	20.41	26.18	23.56±1.24	21.59	26.18	23.50±1.23	21.65	25.97	23.56±1.25	21.59	26.18
ACD	L	3.73±0.24	3.09	4.39	3.72±0.25	3.09	4.10	3.63±0.26	3.09	3.93	3.73±0.24	3.09	4.39
	N	3.76±0.25	2.93	4.55	3.76±0.25	2.93	4.55	3.76±0.25	2.93	4.55	3.77±0.25	2.93	4.55
	H	3.83±0.26	3.23	4.55	3.84±0.26	3.47	4.55	3.88±0.25	3.58	4.55	3.84±0.26	3.47	4.55
CR	L	7.68±0.23	7.08	8.11	7.71±0.21	7.08	8.03	7.67±0.18	7.41	7.94	7.67±0.23	7.08	8.11
	N	7.77±0.25	7.22	8.53	7.76±0.26	6.85	9.61	7.76±0.26	6.85	9.61	7.77±0.26	6.85	9.61
	H	7.80±0.28	6.85	9.61	7.84±0.22	7.34	8.19	7.88±0.22	7.40	8.17	7.84±0.22	7.34	8.19
CP	L	43.99±1.36	41.62	47.68	43.80±1.20	42.03	47.68	44.05±1.00	42.51	45.55	44.00±1.36	41.62	47.68
	N	43.48±1,37	39.57	46.79	43.58±1.42	39.57	49.31	43.56±1.40	39.57	49.31	43.52±1.40	39.57	49.31
	H	43.46±1.45	41.21	49.31	43.06±1.24	41.21	45.99	42.85±1.20	41.35	45.66	43.06±1.24	41.21	45.99
CCT	L	565.45±34.51	511	648	569.12±25.40	511	648	545.25±32.33	511	586	565.45±34.50	511	648
	N	568.82±35.66	479	691	567.34±36.86	465	691	567.86±35.67	465	691	567.76±36.29	465	691
	H	565.96±35.73	465	635	571.84±35.85	497	616	576.74±28.88	525	616	571.43±30.04	497	616
Meas = measurement, Gr = group, L = patients with a low Z-score, N = patients with a relatively normal Z-score, H = patients with a high Z-score, HFAsZ = height for age Z score, SD = standard deviation, Min = minimum, Max = maximum, SER = spherical equivalent refraction, AL = axial length, ACD = anterior chamber depth, CR = average corneal radius, CP = average corneal power, CCT = central corneal thickness													

**Table 5:** The measurement values of L, N, and H for BMIsZ for the values of “1”, “1.5” and “2” and the final group “-1 to +1.5”

		BMIsZ values for ±1			BMIsZ values for ±1.5			BMIsZ values for ±2			BMIsZ values for -1 to +1.5		
Meas.	Gr.	Mean±SD	Min	Max	Mean±SD	Min	Max	Mean±SD	Min	Max	Mean±SD	Min	Max
SER	L	0.65±2.52	-4.00	7.13	-0.64±1.60	-4.00	1.75	-0.98±2.16	-4.00	1.75	0.65±2.52	-4.00	7.12
	N	0.30±2.62	-6.62	7.37	0.49±2.61	-6.65	7.37	0.53±2.54	-6.62	7.37	0.42±2.59	-6.62	7.37
	H	0.58±2.40	-5.00	6.62	0.38±2.38	-4.50	5.75	0.01±2.49	-4.37	5.75	0.38±2.38	-4.50	5.75
AL	L	23.03±1.01	19.91	25.40	23.26±1.06	21.36	25.40	23.59±1.12	22.52	25.40	23.03±1.01	19.91	25.40
	N	23.03±1.20	20.09	26.20	23.00±1.25	19.91	30.92	22.97±1.23	19.91	30.92	23.01±1.26	20.09	30.92
	H	23.00±1.35	20.41	30.92	23.03±1.28	20.42	26.18	23.26±1.33	20.42	26.18	23.03±1.28	20.42	26.18
ACD	L	3.76±0.24	3.09	4.12	3.85±0.18	3.48	4.12	3.91±0.13	3.71	4.12	3.76±0.24	3.09	4.12
	N	3.76±0.24	3.08	4.55	3.76±0.25	2.93	4.55	3.76±0.25	2.93	4.55	3.76±0.25	2.93	4.55
	H	3.78±0.27	2.93	4.55	3.80±0.25	3.19	4.55	3.82±0.26	3.19	4.55	3.80±0.25	2.93	4.55
CR	L	7.77±0.30	7.32	8.53	7.63±0.18	7.32	7.84	7.66±0.13	7.44	7.83	7.77±0.30	7.32	8.53
	N	7.76±0.24	7.08	8.43	7.78±0.26	6.85	9.61	7.76±0.26	6.85	9.61	7.77±0.25	6.85	9.61
	H	7.76±0.25	6.85	9.61	7.74±0.24	7.24	8.37	7.78±0.24	7.32	8.37	7.74±0.24	7.24	8.37
CP	L	43.48±1.62	39.57	46.11	44.25±0.92	43.06	46.11	44.09±0.73	43.14	45.36	43.48±1.62	39.57	46.11
	N	43.53±1.39	40.07	47.68	43.48±1.42	39.57	49.31	43.57±1.41	39.57	49.31	43.52±1.38	40.07	49.31
	H	43.59±1.37	40.32	49.31	43.65±1.35	40.35	46.65	43.43±1.33	40.35	46.16	43.66±1.35	40.35	46.65
CCT	L	568.61±25.40	497	606	559.72±34.68	497	606	568.00±51.75	497	606	568.61±255.40	497	606
	N	567.14±36.86	479	691	567.73±35.07	479	691	567.11±34.35	479	691	567.24±36.03	479	691
	H	568.67±35.85	465	642	569.20±36.94	465	640	571.84±40.49	465	640	569.20±36.94	465	640
Meas = measurement, Gr = group, L = patients with a low Z-score, N = patients with a relatively normal Z-score, H = patients with a high Z-score, BMIsZ = body mass index for age Z score, SD = standard deviation, Min = minimum, Max = maximum, SER = spherical equivalent refraction, AL = axial length, ACD = anterior chamber depth, CR = average corneal radius, CP = average corneal power, CCT = central corneal thickness													

**Table 6:** The statistical comparison of the measurement values between the groups for a value of “1”, “1.5” and “2” and the final group “-1 to +1.5”

Meas.	Compared groups	P value for ±1 HFAsZ	P value for ±1,5 HFAsZ	P value for ±2 HFAsZ	P value for -1 to +1.5 HFAsZ	P value for ±1 BMIsZ	P value for ±1,5 BMIsZ	P value for ±2 BMIsZ	P value for -1 to +1.5 BMIsZ
SER	L- N	1.000	1.000	1.000	0.679	1.000	0.155	0.277	1.000
	H - L	0.006	1.000	1.000	0.172	1.000	0.266	0.855	1.000
	H - N	*0.002	0.421	1.000	0.578	0.470	1.000	0.117	1.000
AL	L- N	0.104	1.000	0.863	0.044	1.000	1.000	0.480	1.000
	H - L	*<0.001	*0.005	0.120	*<0.001	1.000	1.000	1.000	1.000
	H - N	*0.001	*0.001	0.150	0.002	1.000	1.000	0.062	1.000
ACD	L- N	1.000	0.660	0.119	0.740	1.000	0.318	0.284	1.000
	H - L	*0.012	*0.024	*0.006	0.019	1.000	1.000	1.000	1.000
	H - N	*0.011	*0.048	0.056	0.049	1.000	0.207	*0.037	0.299
CR	L- N	*0.001	0.391	0.512	0.002	1.000	*0.036	0.789	1.000
	H – L	*0.001	*0.010	*0.034	<0.001	1.000	0.192	0.578	1.000
	H - N	1.000	*0.037	0.058	0.008	1.000	0.345	1.000	0.572
CP	L- N	*0.002	0.856	0.602	0.004	1.000	0.046	0.894	1.000
	H – L	*0.009	*0.012	*0.031	<0.001	1.000	0.203	0.607	1.000
	H - N	1.000	*0.011	*0.036	0.028	1.000	0.471	1.000	0.760
CCT	L- N	1.000	1.000	0.221	1.000	1.000	1.000	1.000	1.000
	H – L	1.000	1.000	0.106	1.000	1.000	0.862	1.000	1.000
	H - N	0.871	1.000	0.851	1.000	1.000	1.000	0.779	1.000
Meas = measurement, L = patients with a low Z-score, N = patients with a relatively normal Z-score, H = patients with a high Z-score, HFAsZ = height for age Z score, BMIsZ = body mass index for age Z-score, SER = spherical equivalent refraction, AL = axial length, ACD = anterior chamber depth, CR = average corneal radius, CP = average corneal power, CCT = central corneal thickness. *Statistically significant values are indicated in bold.									

## Discussion

In this prospective observational case series, the effect of anthropometric measurements on ocular biometric changes was investigated using the Z-scoring system. In addition, in patients with significant ocular biometric changes, an attempt was made to determine an anthropometric measurement value corresponding to these values. In this way, patients were grouped according to their Z-score values as patients with a low Z-score (L), patients with a relatively normal Z-score (N), and patients with a high Z-score (H); and biometric differences between these groups were evaluated statistically. In subjects (patients) with low and high Z-scores, Z-score was more significant in biometric changes; negative and positive 1, 1.5, and 2 values and afterward. In light of these values, -1 and +1.5 values were determined as the cut-off.

In the grouping created for the value of “1” for HFAsZ, H had significantly lower SER, longer AL, and longer ACD than other patients. Also, L had significantly lower CR and higher CP than other patients. However, for SER, AL, and ACD, the differences between L and N were insignificant. In addition, for CR and CP, no statistically significant difference was determined between H and N.

When groups were rearranged for the value of “2” for HFAsZ, the difference in AL and SER values between groups was completely insignificant. Whereas there was a significant difference between L and H in ACD and CR, there was no significant difference between these patients with N. In terms of CP, H had a significantly lower CP value, while there was no significant difference between L and N, unlike the grouping designed according to HFAsZ for the value of “1”. In fact, in terms of CP, groups designed according to values “1” and “2” presented similar results, albeit in different ways. For the value of “2” for HFAsZ, these results were not satisfactory enough. 

Although no significant results could be obtained for SER in the grouping designed for HFAsZ for the value of “1.5”, almost similar results were collected for AL and ACD with the grouping designed according to the “1” value. However, significantly higher CR and lower CP values were determined in H. Indeed, for CP and CR, these results were parallel to the findings in HFAsZ for the value of “1” in a different way.

To combine the results in HFAsZ for the value of “1” and “1.5” groupings, after rearrangement of patients with a Z-score lower than -1 as L, those with a Z-score over +1.5 as H, and other relatively normal Z-scores as N, the results were more striking. Although no significant result could be obtained in terms of SER, there were significant differences in all groups in terms of AL, ACD, CR, and CP. From L to N and H, AL and CR increased significantly in each patient group, while CP decreased significantly. While there was an increase in ACD from L to N and from there to H, a statistical significance was observed between N and H.

All the results for CCT were insignificant in all the groups.

In their study, Hashemi et al. [**21**] determined that AL was positively associated and SER had a negative correlation with height. For the values of -1 to +1.5, AL results in HFAsZ were in line with this study. Ye et al. [**22**] found a significant positive correlation between height with AL and ACD and a significant negative correlation with SER. These results support the results of our study except for SER. No significant relationship was found between HFAsZ and CCT in this present study, on the other hand, a significant positive correlation was found between height and CCT in the study of Ye et al. [**22**], but this correlation was weak (r=0.17, p<0.01). Although there was no relationship between height and CR in the same study [**22**], in the present study, CR increased significantly from L to N and from there to H. Similar to our results, in a cross-sectional study of 1449 Chinese school children, the eyeball length in children in the fourth quartile was 0.46 mm longer, the CR of curvature was 0.10 mm greater (i.e., flatter), and refraction was more negative by 0.47 diopters [**23**].

In this study, no significant relationship was determined between BMIsZ and any ocular biometric measurement. Similar to our study, in the study of Ojaimi et al. [**24**], no significant trends were observed in all ocular biometric parameters or SER by quintiles of weight, BMI, body fat percentage, or waist circumference. Also, Wong et al. [**25**] studied Singapore Chinese adults and found that weight and BMI did not affect the ocular parameters in their study. In the study of Hashemi et al. [**21**] that investigated the association of SER with ocular biometry components adjusted for demographic variables in children aged 6-12 years with multiple linear regression, no relationship was determined between weight and SER. However, contrary to these results, Ye et al. [**22**] found a significant correlation between weight and SER, AL, CCT ACD, and CR. In the present study, it was believed that investigating the relationship between weight and ocular biometric measurements might not have been a very accurate approach. It is an undeniable fact that as the development of children continues, their weight will increase with their height. Even in our study, there was a strong correlation between the children’s heights and weights. For this reason, it is not surprising that 2 parameters with such a high correlation result were found with similar statistical findings. In the mentioned study, it was thought that the similar correlation coefficients of height and weight with ocular biometrics were the reason for these results.

In the present study, significant differences were determined between male and female children in terms of AL, ACD, CR, CP, and CCT. Males had a more astigmatic error, longer AL and ACD, flatter corneas, less CP, and higher CCT. In a study that explored the conveyance of ocular biometric parameters and refraction in a population-based study of Australian children, supporting our study, mean AL was longer, ACD was higher, and the cornea was flatter in the boys [**26**]. In their study, Huang et al. [**27**] also stated that boys showed a significantly longer AL and a greater CR, which was parallel to our study. Similarly, IP et al. [**28**] reported in their study that boys had slightly flatter corneas, longer eyes, and deeper chambers than girls. In the study of Zhang et al. [**29**] boys had longer AL and greater CR. A recent study also found that girls had significantly thinner corneas than boys by an average difference of approximately 5 μm [**30**]. This result contradicts the present study. On the other hand, in the study of Gul et al. [**31**] supporting the current study, there was only a 6-µm CCT difference between boys and girls, which was not statistically significant. In the same study, AL and ACD were significantly higher in boys, which is logical and parallel with the present study. 

In this present study, the relationship between ocular biometrics and anthropometric measurements was investigated. Z-score was used for height and BMI for anthropometric measurements. The relationship and contact between anthropometric measurements and ocular biometrics have also been investigated in previous studies, but to our information, the Z-score has not been used before on this subject [**22**-**24**]. Based on the hypothesis that the factors affecting growth and development in the whole body in children may also affect the ocular structures, it is thought that anthropometric values including numerical and objective results such as Z-score can help us to understand these effects better. For this reason, if the Z-score is desired to be used as a tool to predict the changes in biometric data, according to our findings it may be wiser to take -1 in the negative direction and +1.5 in the positive direction as the limit value instead of the values of “1”, “1.5” or “2”.

## Conclusion

A limitation of our study was the moderately small sample size. Studies with larger sample sizes can offer more valuable information. One of the foremost important viewpoints of our study was that it is the first consideration to examine the relationship between Z-score and ocular biometry and to propose a reference value in this regard.

**Conflict of Interest Statement**

The authors declare that they have no conflict of interest.

**Informed Consent and Human and Animal Rights Statement**

Informed consent was obtained from all the parents and legal guardians of the individuals included in this study.

**Authorization for the use of human subjects**

Ethical approval: The research related to human use complied with all relevant national regulations and institutional policies, followed the tenets of the Helsinki Declaration, and was approved by the Ethics Committee of Beylikdüzü Public Hospital, Istanbul, Turkey (2057/22.11.2019).

**Acknowledgments**

None.

**Sources of Funding**

None.

**Disclosures**

None.
